# The Importance of Early Recognition of Tuberculosis—Its Curability with Special Reference to Climatology

**Published:** 1894-10

**Authors:** T. W. Conerly

**Affiliations:** San Angelo, Texas


For Texas Medical Journal.

### THE IMPORTANCE OF EARLY RECOGNITION OF TUBERCULOSIS.

### Its Curability With Especial Reference to Climatology.

BY T. W. CONERLY, M. D., SAN ANGELO, TEXAS.

### THE CONCHO COUNTRY.

ON THE early recognition of tuberculosis depends in a large measure its proper and successful management.

The old opinion that tuberculosis is incurable can no longer be maintained; however, cures are the exception and fatalities the rule. In the early stages of the disease the primary deposit is quite small, and at this time the resisting power of the patient is greater than ever afterwards. Were we so fortunate as to make our diagnosis at this stage, many more cases might recover, but in practice we are not likely to meet these patients until constitutional symptoms appear, and then but a small per cent. of cures are effected.

In order to be able to recognize tuberculosis early it is necessary to bear in mind that the primary seat of the disease may occur in any organ or tissue which has a blood supply, and that the disease may exist without constitutional symptoms.

The first place to look for the disease is of course the lungs, as that is the most frequent seat.

"In examining the lungs for tubercle, one must not expect pronounced indications in the early stage. The two places in the lungs in which one will most frequently find the primary deposit are the apices and bronchial glands. When the primary deposit takes place in the apices, if the deposit is quite small, both the climical and physical symptoms will be very meager; there may be scarcely any cough, and the physical signs of all except the extreme apices of the lungs will be normal. By careful examination over and above the clavicle, in front and along the upper border of the scapula behind, one will find such signs of deposit as may exist. A deposit, be it ever so small, is bound to impair the resonance and produce a prolonged expiratory sound. The first of these signs is always present, and can be detected by an acute and skillful ear, but the latter will be absent upon shallow respiration, and can only be heard upon an inspiration deep enough to fill the air cells of the affected party."*

When the primary deposit is made in the bronchial glands, which is the usual seat when the bacillii find their way into the system through the respiratory tract, it is very difficult to make an early diagnosis, perhaps the most prominent symptom being a hacking cough. As soon as the first glands break down, which process is indicated by beginning expectoration, the microscope will reveal the true nature of the trouble.

M. Aubrey, an eminent French clinician, states that by a microscopical examination of the blood, milk, spermatic and other fluids, he was able to diagnose tuberculosis before the usual symptoms presented, and in fact, before a suspicion of tuberculosis existed.

With a good microscope possessing accessories of not less than 600 diameters and a little practice in staining, one can at least examine the sputum, and will be able to make his diagnosis positive, as well as satisfactory to himself.

The great importance of an early diagnosis is emphasized by the fact that in the dead house, and at the post-mortem exami-

---

*University Medical Magazine, Nov., 1893.

nation cases of healed tubercular foci are found in the lungs of subjects who have died of other trouble. Quite a lot of statistics bearing upon this subject have accumulated during recent years; some of these published in the *Review* are as follows:

Thos. Vibert found that of 131 persons meeting sudden or violent death whose bodies were brought to the Paris morgue, 17 showed evidence of cured tuberculosis. Sidney Martin found 42 cases of healed tuberculosis of the lungs in 445 post-mortem examinations. Heighler found in 4 per cent. of his autopsies evidences of healed tuberculosis. H. P. Loomis found in 763 persons dying of a non-tubercular disease 71, or 9 per cent., which presented pulmonary changes characteristic of healed tuberculosis.

These statistics would indicate that pulmonary tuberculosis is quite amenable to arrest and cure much more frequently than the opinion of the general prrctitioner would seem to indicate.

From the same article I quote as follows:

"The walls of healed cavities consist of thick, dense, pigmented fibrous tissue, and the more or less retracted space may or may not communicate with a bronchus, usually there are pleuritic adhesions and puckering in the pleural surface of the lung over the healed tubercular district, which in the vast majority of instances is found in the apex. It was formerly thought that in cases of healed tubercular foci, that the healed area was free from tubercular bacilli. Loomis however found that in three out of twelve cases of apparently arrested tuberculosis, inoculation experiments with rabbits showed that bacilli or their spores were still present in the tissues.

Therefore, it is plain that the disease may remain in a latent state for an indefinite length of time, but it is possible that bacilli might escape from those areas and make their way to different parts of the body and acute tubercular foci be thus set up. It is to be impressed, however, from the investigations of Loomis that in the majority of such cases the bacilli either become extinct or fail to escape from the tissues surrounding the primary foci.

After a careful study of the results obtained from these investigations and several years of personal observation, my conclusions are that the disease is curable in a large percentage of cases in the easier stages of the disease, and occasionally in the more advanced stages.

During the last few years tuberculosis has received more attention and investigation from the medical profession and the medical press than has any other disease.

The materia medica has been culled and sifted in an endeavor to arrive at suitable and reliable therapeutics for tuberculosis. Cod liver oil, hypophosphites, creasote, iron and various tonic measures have been resorted it, but cures by these agents are so few that they have come to be regarded only as helps to bridge over an emergency, as splints to hold together a deplorable system of patchwork. These results and these facts serve as arguments why those who expect or desire more than temporary alleviation should make an immediate and permanent change of residence.

The necessary requirements for any climate to possess in which patients with chronic pulmonary affections may expect to obtain permanent benefit, and in which all students of climatology agree, are well set forth in the following synopsis, drawn up by Dr. Charles Denison, of Denver, Colorado:*

Dryness as opposed to moisture.
Coolness as opposed to warmth or heat.
Rarefaction as opposed to sea-level pressure.
Sunshine as opposed to cloudiness.
Variability as opposed to equability.

These various requirements may be found combined in a vast scope of our country, in New Mexico, Colorado, Arizona, a part of California, and ideally so in a large part of Western Texas. Arizona, and the mountainous districts of California, have some reputation in this direction, but New Mexico and Colorado have perhaps recently advertised their climate more extensively than any other State or country, and they are excellently suited to patients who are yet in fair physical vigor, and have not a tendency to pulmonary hemorrhage. But many patients, and some physicians, commit the error of thinking that if a little altitude is good, a great deal of altitude is better, and a patient is sent at once from a warm climate of equability of temperature and a high degree of sea level pressure, to an altitude of 4000 to 6000 feet, with its rarefied atmosphere. The change is usually too great, and often an alarming pulmonary hemorrhage takes place, and the patient must be hurried back, a worse invalid than be-

---

*Reprint of paper read before the ninth International Medical Congress, 1887, "The preferable climate for consumptives."

fore. These most prominent objections are very happily eliminated from the factors that go to make the climate of Western Texas. It is not always the case that the same climatic conditions and the same altitude will suit all patients alike. Some cases will do best at a greater altitude, with its corresponding rarefaction, than others will. But the requirements of any case may be met in the scope of country lying between the Colorado river and the Rio Grande, at an elevation of 1500 to 3500 feet, between Runnels and Concho counties and the Devil's river. One hundred miles southwest, perhaps, is situated the most desirable part of this State as a health resort for tuberculous individuals. The picturesque and fascinating Concho valley is here situated, with its river of the same name, whose clear crystal waters go charging over rapids and rushing to the sea. The average elevation of this scope of country is 2000 to 2500 feet, and includes several nice towns, where one may live and be provided for in any style that may suit his individual fancy or requirements. Ballinger is situated on the Colorado river, and also on the Gulf, Colorado & Santa Fe Railroad; San Angelo is at the terminus of the same railroad, on the banks of the beautiful Concho river; Sherwood, Sonora, Ozona, and a number of nice smaller towns, are all reached by daily stage from San Angelo. The country is sparcely settled, many large ranches occupying a greater part of it. But little farming is attempted, except along the water courses, where irrigated.

The hygroscopic condition of the atmosphere is that of extreme dryness. Dews seldom fall at any season of the year. Many of the people sleep out of doors in summer, with perfect immunity from any evil effects, as would occur from such exposure in lower and more sultry localities. The Concho valley is near enough to the gulf to get the benefit of the atmospheric movement coming from that source, but so far away that the moisture is eliminated, and the sweeping gale from the gulf is moderated before reaching this locality.

A slight objectionable feature here is the dust that is sometimes blown before the wind; but this objectionable feature is to be met with in all dry localities where there is considerable atmospheric movement, and is as seldom seen here as in any dry country, and is found to be of but little deleterious effect to pulmonary affections. The days in summer that are quite warm have this discomforting feature very much modified and offset by the pure and refreshing breeze that is constantly wafted over mountain and through valley.

The summer nights are remarkable for their recuperating effect; one gets out from under cover every morning, feeling much refreshed and invigorated. In winter, the weather is variable. Some very cold, life-giving days occur, with the wind pouncing down from the north; it is never the dreaded blizzard, but a healthful, bracing temperature, which is quite easily endured, and lasts but a few days. Ninety-five per cent. of our winter weather is mild and balmy, o'er spread by a sky that is simply Italian in its beauty.

Some patients find it beneficial to camp out. This practice is to be encouraged when the weather is fine and all necessary comforts are provided, but by no means would I advise it, when its practice would debar one of the means of comfort and pleasure; ''roughing it'' is not to be countenanced. Many people sleep on their open galleries all summer, year after year, quaffing the pure ozone from the gentle zephyrs that never fail us.

This city and county are getting to be quite a resort for persons suffering with all kinds of lung affection. A number have made their way here who had visited many other localities, and found them unsuited to their condition, and have improved and recuperated to a remarkable extent here, within a few months. There are hundreds of healthy persons in this country to-day who a few years ago were suffering from chronic bronchitis, incipient tuberculosis, or unresolved pneumonia. Some invalids come here who are so low as to be confined to bed, and are carried to their house or hotel on a stretcher, who recuperate and get up; but as a rule, this latter class have come too late to be even temporarily benefited.

The roads over the Concho valley are, for the most part, very good. Buggy and horeback riding are a great source of recreation, and the bicycle is used very largely by both sexes.

San Angelo, though a small place, is well prepared to take care of health seekers. The city population is about 4000; has an excellent system of water-works, obtained from the pure, sparkling Concho river; an ice factory, fresh vegetables and fresh fish at all seasons of the year, ample hotel and boarding house accommodation, as well as cottages to be had on quite low rental. We have seven churches, an open hall, and an elegantly fitted up club room, etc.

